# Lanthanum hexaboride for solar energy applications

**DOI:** 10.1038/s41598-017-00749-w

**Published:** 2017-04-06

**Authors:** Elisa Sani, Luca Mercatelli, Marco Meucci, Luca Zoli, Diletta Sciti

**Affiliations:** 1 CNR-INO National Institute of Optics, Largo E. Fermi, 6, I-50125 Firenze, Italy; 2 CNR-ISTEC, Institute of Science and Technology for Ceramics, Via Granarolo 64, I-48018 Faenza, Italy

## Abstract

We investigate the optical properties of LaB_6_ – based materials, as possible candidates for solid absorbers in Concentrating Solar Power (CSP) systems. Bulk LaB_6_ materials were thermally consolidated by hot pressing starting from commercial powders. To assess the solar absorbance and spectral selectivity properties, room-temperature hemispherical reflectance spectra were measured from the ultraviolet to the mid-infrared, considering different compositions, porosities and surface roughnesses. Thermal emittance at around 1100 K has been measured. Experimental results showed that LaB_6_ can have a solar absorbance comparable to that of the most advanced solar absorber material in actual plants such as Silicon Carbide, with a higher spectral selectivity. Moreover, LaB_6_ has also the appealing characteristics to be a thermionic material, so that it could act at the same time both as direct high-temperature solar absorber and as electron source, significantly reducing system complexity in future concentrating solar thermionic systems and bringing a real innovation in this field.

## Introduction

Lanthanum hexaboride (LaB_6_) is a well known thermionic material (see for instance refs 1–5 among the many available). Its ability to emit electrons has been widely exploited in last 60 years since the pioneering work of Lafferty on sintered hexaboride ceramics^6^. It has been used to build hot cathodes for a large variety of applications, including electron guns in cathode tubes^7^, cathodes for plasma production^8, 9^ and electron microscopes^10, 11^. While at present, mainly single crystalline emitters are used, sintered ceramics show thermionic properties as well, and historically they have been the first to be studied^6, 7, 12^. It has been shown that, if surface impurities are removed, sintered LaB6 can show a work function value of 2.36 eV, which is fully comparable to that of single crystals (2.28 ÷ 2.47 eV)^13^. In addition, photo-induced electron emission is documented in the literature both by single crystals and by polycristalline specimens^14, 15^, as well as simultaneous thermionic emission and photoemission under excitation of a polycrystalline film at 442 nm wavelength (2.8 eV)^16^.

In the field of material processing, LaB_6_ is also used as additive to other diboride ultra-high temperature ceramics (UHTCs) for improving their oxidation resistance at extreme temperatures^17, 18^. Because of its well-defined plasma reflection edge making it a textbook case, optical properties of bulk and thin film LaB_6_ have been investigated and theoretically modeled in the past^19–21^. Recently, the attention on optical properties of LaB_6_ has been renewed, but to the best of our knowledge, it is mainly restricted to the use of this material in form of nanoparticles^22–24^.

Renewable energies, and in particular solar energy exploitation issues, are a key topic for the future. At present, the main technologies for concentrating solar systems are solar thermal power and concentrating photovoltaics. However, very recently a novel approach has been proposed for the direct generation of power from concentrated sunlight exploiting both thermionic and thermoelectric effects^25^. In the cited paper, the solar absorber and the thermionic emitter were different materials, with the thermionic emitter (a thin diamond film) deposed on the back surface of the sunlight absorber (an UHTC carbide with a special treatment to increase solar absorbance).

From our previous studies on UHTC carbides and borides^26–34^, we have assessed strength and weakness points for the use of this class of novel materials as innovative high-temperature solar absorbers in thermodynamic solar plants. In fact, the ideal solar absorber should withstand very high temperatures while maintaining, with no damage, good mechanical strength and stability, high thermal conductivity, high solar absorbance to efficiently collect sunlight, and low emittance at operating temperatures, to minimize unwanted energy losses by thermal re-radiation^35–37^. From point of view of optical properties, strength points of UHTC materials we have investigated so far are the intrinsic low thermal emittance and good spectral selectivity, while solar absorbance typically is to be improved, e.g. by proper surface treatments^38, 39^. In this work we report on the microstructural and optical characterization of LaB_6_ ceramics. For thermodynamic solar applications, the knowledge of optical properties of LaB_6_ is of interest because, as recalled above, it is used as additive for UHTC borides.

**Figure 1:**
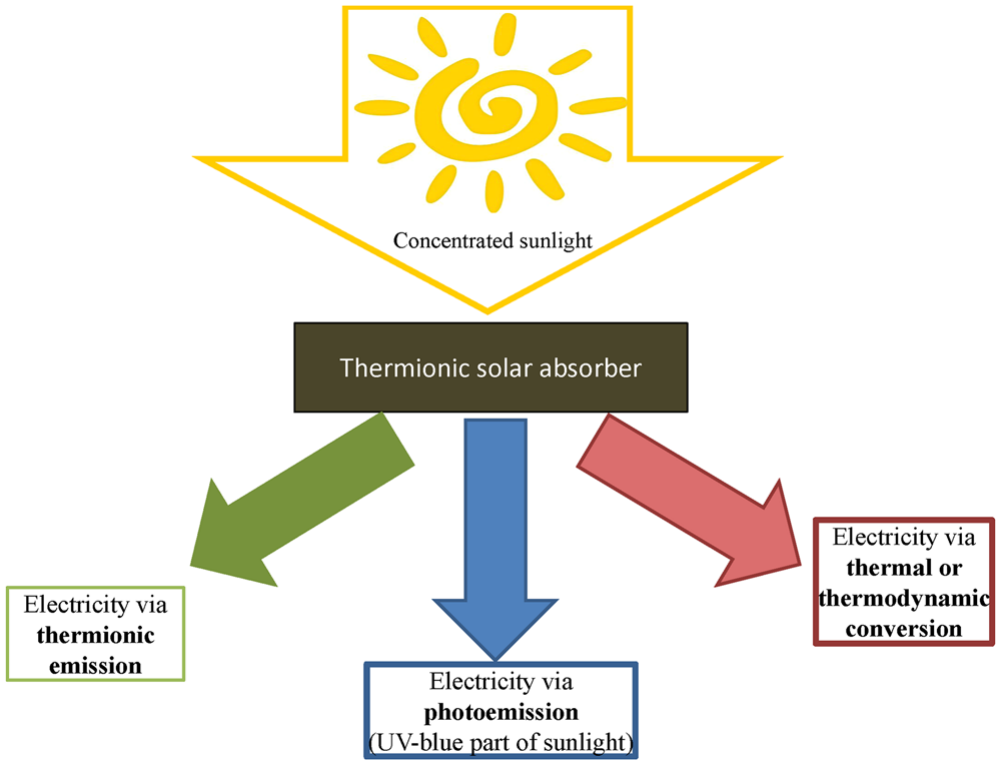
Concept of the thermionic/thermodynamic solar absorber.

However, we point out that the innovation potential of LaB6 is much higher. In fact, compared to previously investigated UHTCs, LaB_6_ has the appealing characteristics to be a thermionic material, so that it could act at the same time both as direct high-temperature solar absorber and as electron source, significantly reducing system complexity in future concentrating solar thermionic systems and bringing a real innovation in this field. Moreover, if surface impurities will be carefully controlled, e.g. with a proper control of production processes and with operation in vacuum, so that the work function is maintained below 4.13 eV (corresponding to light wavelengths longer than 300 nm), direct electron emission (photoemission) from LaB_6_ induced by absorption of the UV-blue spectral portion of concentrated sunlight could be additionally and simultaneously exploited (Fig. 1). Thus, to assess the potential of LaB_6_ for this radically new concept of solar absorber we carried out our study as a function of the sample porosity and surface finishing.

## Results and Discussion

### Microstructural features

**Figure 2:**
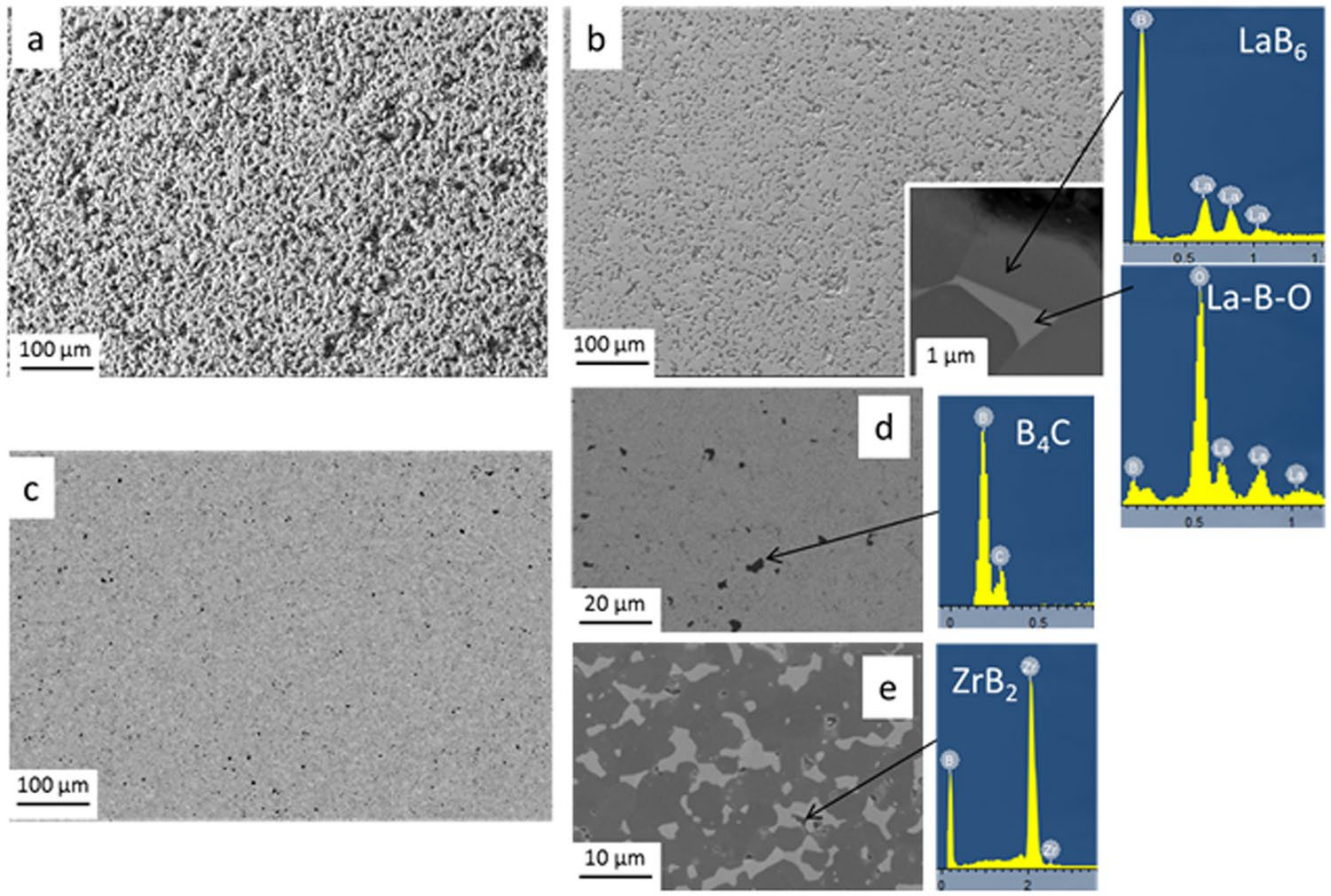
(**a**) Rough surface of porous LaB_6_ in SE imaging, (**b**) polished surface of porous LaB_6_ in SE imaging, Inset: high magnification of porous LaB_6_ microstructure showing oxide phases (La_2_O_3_) wetting the grain boundaries and relative EDS spectra, (**c**) polished surface of dense LaB_6_ in SE imaging, (**d**) higher magnification details showing B_4_C pockets in BSE imaging; (**e**) ZrB_2_ inclusion in LaB_6_ matrix in-lens imaging (**d**) and corresponding EDS spectra.

Table 1 lists investigated samples, while Fig. 2 shows microstructures and EDS spectra. As expected, the monolithic material (LaB_6__p), Fig. 2a,b contains a significant fraction of open porosity, in agreement with density measurements. The grain size ranges from 3 to 10 µm. Traces of La-B-O spurious phases were recognized along the grain boundaries by SEM-EDS analysis. (see inset of Fig. 2b and corresponding EDS spectra). These impurities are typical contaminants of the boride powders and hindered the material densification during hot pressing^40^.

**Table 1:** List of materials, compositions, densities and surface roughness.

Sample label	Composition Vol%	Description	Bulk density, relative density		Ra, Rt (µm)
			g/cm^3^	%	
LaB6_p rough	100% LaB_6_	Porous, as sintered	3.7	80%	0.58 ± 0.06 4.70 ± 0.83
LaB6_p polished	100% LaB_6_	Porous, Polished up to 1 µm	3.7	80%	0.09 ± 0.01 0.52 ± 0.09
LaB6_d polished	LaB_6_ + 15 vol% ZrB_2_ + 3 vol% B_4_C	Dense, Polished up to 1 µm	4.86	100%	0.03 ± 0.01 0.19 ± 0.05

The letter _p in the name stands for porous, while the suffix _d identifies the dense sample. *R*_a_: mean surface roughness; *R*_t_: distance between the highest asperity and the lowest valley.

From both Fig. 2a,b and Table 1 the difference of surface roughness between as sintered and polished surface can be appreciated (Ra decreased from 0.58 to 0.09 µm for unpolished and polished surfaces, respectively).

As for the LaB_6_ _d sample, addition of ZrB_2_ and B_4_C notably improved the densification. The final material was completely dense and the microstructure can be observed in Fig. 2c–e. According to XRD (not shown) no extra phases formed. Dark spots in Fig. 2c,d belong to B_4_C, while the in secondary electron imaging LaB_6_ and ZrB_2_ display similar contrast. By in-lens signal, see Fig. 2e, dark contrasting grains belong to LaB_6_, whilst light contrasting grains belong to ZrB_2_. The improvement in the densification was mainly attributed to the addition of B_4_C, via cleaning of surface oxides such as La_x_O_y_, B_x_O_y_ from the boride particles, as observed for similar ceramics^40^. Increase in the final density also affected the quality of surface polishing and consequently the final roughness decreased to Ra 0.03 µm, see Table 1.

### Optical characterization

**Figure 3:**
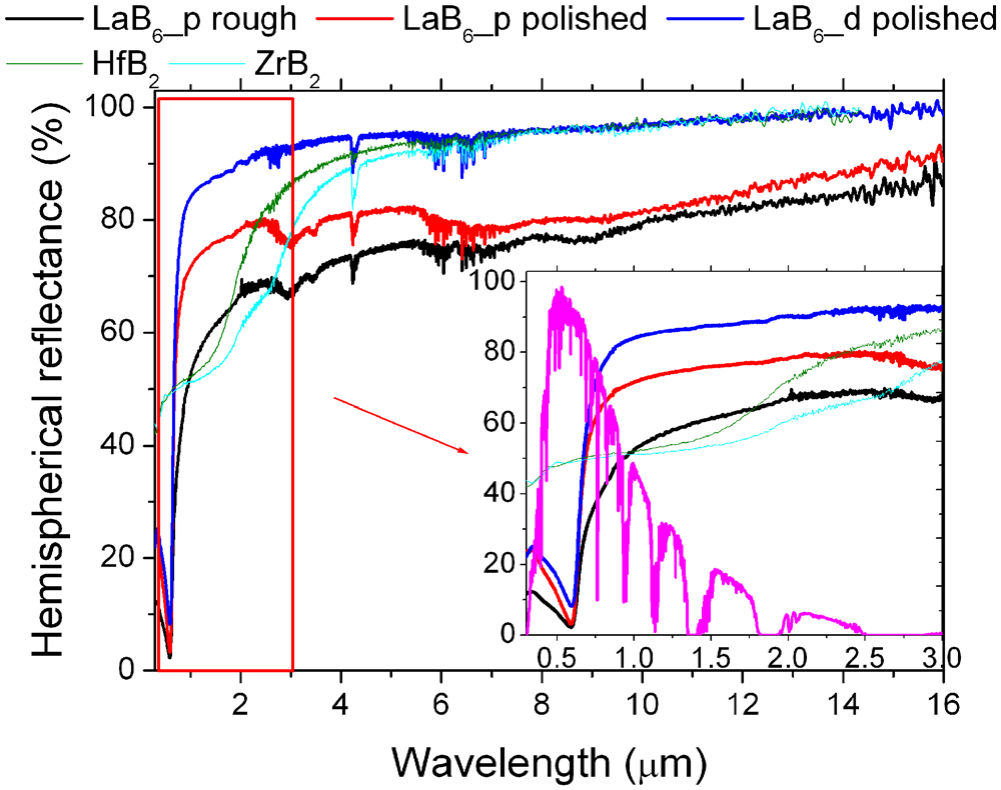
Reflectance spectra of samples, compared to dense samples of hafnium and zirconium borides from ref. 41.

Figure 3 shows the reflectance spectra of LaB_6_ samples, compared to previously analyzed dense hafnium and zirconium borides^41^. The inset of Fig. 3 shows the reflectance in the sunlight spectral region, the sunlight spectrum is also superimposed for reference.

LaB_6_ reflectance directly increased with decreasing of the surface roughness. This is particularly evident comparing LaB_6__p rough and LaB_6__p polished. For the dense composite material, the reflectance was further improved due to the almost complete elimination of porosity. The spectrum shape presents very similar features in pure LaB_6_ and the LaB_6_ –based composite containing ZrB_2_ and B_4_C, except for a local minimum at round 3 μm wavelength and a couple of secondary small minima between 7 and 10 µm, present in both spectra of porous samples. These features could be ascribed to oxide phase impurities in porous LaB_6_ ceramics, like La-O phases, B-O, or mixed La-B-O phases (as those observed in Fig. 2b). In contrast, these spurious phases were not observed in the optical spectra of LaB_6_ _d owing to their removal by B_4_C, as previously mentioned. No specific features in the spectrum that could be attributed to B_4_C inclusions were recognized in LaB_6__d.

Compared to hafnium and zirconium borides, LaB_6_ has a considerable lower reflectance (i.e. a higher absorbance) near the peak of sunlight spectral distribution. However, thanks to peculiar spectral features of LaB_6_, the rough porous sample, LaB_6__p rough remains more absorptive up to about 1 μm wavelength. Towards longer wavelengths, the reflectance of porous pellets is about 20% lower than that of other borides, while the dense sample shows a similar reflectance at the infrared plateau region.

**Figure 4:**
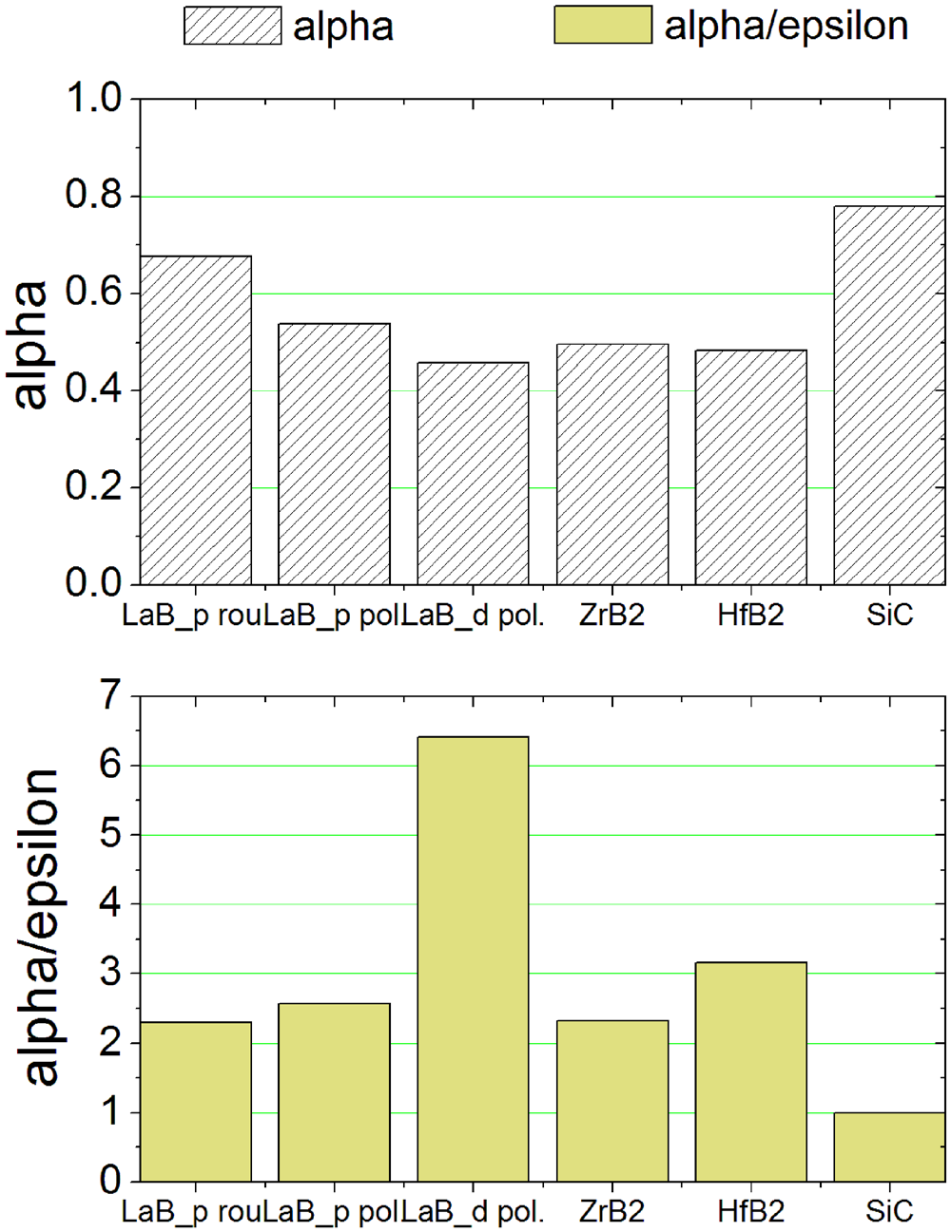
Calculated solar absorbance α and solar selectivity α/ε for LaB_6_ samples and comparison with HfB_2_, ZrB_2_ and SiC.

From the experimental room-temperature hemispherical reflectance ρ^∩^(λ) we calculated the total solar absorbance, α:

1 $$\alpha =\frac{{\int }_{{\lambda }_{\min }}^{{\lambda }_{\max }}(1-{\rho }^{\cap }(\lambda ))\cdot S(\lambda )d\lambda }{{\int }_{{\lambda }_{\min }}^{{\lambda }_{\max }}S(\lambda )d\lambda }$$

where S(λ) is the Sun emission spectrum^42^ and the integration is carried out between λ_min_ = 0.3 µm and λ_max_ = 3.0 µm; and an estimated hemispherical emittance, ε, at 1100 K:

2 $$\varepsilon =\frac{{\int }_{\lambda 1}^{\lambda 2}(1-{\rho }^{\cap }(\lambda ))\cdot B(\lambda ,1100K)d\lambda }{{\int }_{\lambda 1}^{\lambda 2}B(\lambda ,1100K)d\lambda }$$

where B(λ, 1100 K) is the blackbody spectral radiance at 1100 K temperature and λ1 = 0.3 µm and λ2 = 16.0 µm. The α/ε ratio (sometimes called spectral selectivity) is a parameter assessing the material potential for solar receiver applications, and ideally should be taken as high as possible. Figure 4 shows absorbance and spectral selectivity for the investigated LaB_6_ samples, together with the two reference borides.

From Fig. 4 we can immediately appreciate the high potential of LaB_6_ as solar absorber material: both solar absorbance and spectral selectivity are comparable or higher than those of comparison materials ZrB_2_ and HfB_2_^41^. Even more, the rough porous sample (LaB_6__p rough) shows a remarkable solar absorbance of 0.7, which is only slightly lower than that of the most advanced solar absorber material to date in actual plants, namely silicon carbide (SiC, α ≈ 0.8^41^) (see ref. 43 and references therein). As for spectral selectivity, for all samples it remains higher than that of SiC (α/ε ≈ 1^41^) and for most of them it is comparable to that of the two reference borides, with the LaB_6_ dense polished sample showing a remarkable value of α/ε = 6.4. If we compare LaB_6_ pellets each other, we can say that a high solar absorbance is obtained at the expense of spectral selectivity. However, even in its less absorptive form (dense polished), LaB_6_ results promising, as said before, with respect to previously investigated borides, as it shows a comparable absorbance and a remarkably higher spectral selectivity.

For a more reliable assessment of LaB_6_ potential for solar absorber applications, we measured the thermal emittance at high temperature. In fact it is known that emittance calculated from room-temperature reflectance spectra are a very useful tool for a preliminary material evaluation, but underestimate the value at high temperature^44^.

**Figure 5:**
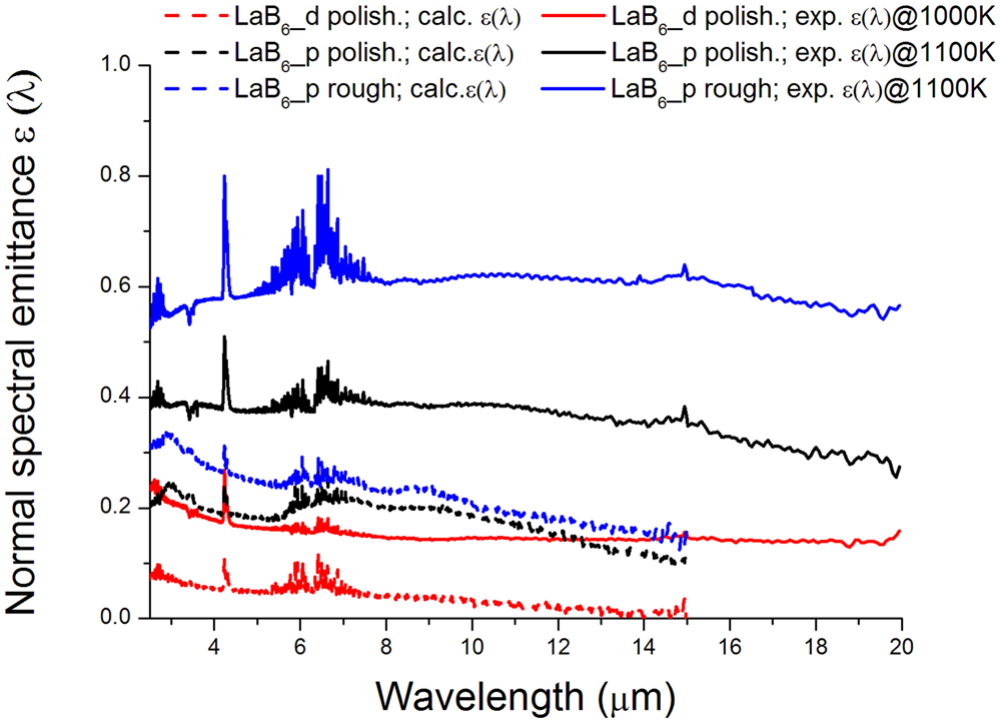
Calculated (dashed lines) and experimental emittance (solid lines) of LaB_6_ samples. The measurement temperature for each sample is indicated in legend. The features at around 3.4 µm, 4.2 µm, between 4.8 and 7.7 µm and around 15 µm are instrumental artifacts due to unbalanced molecular absorption by ambient air in the optical path of the beams outside the furnace.

Figure 5 compares the experimental spectral normal emittances (solid lines) with the values calculated from room-temperature reflectance data ε_calc_(λ) = 1 − ρ^∩^(λ) (dashed values). As previously mentioned, the measured emittance is higher than the calculated one. Samples maintain the hierarchy among them, as expected, with the dense pellet showing the lowest emittance (spectrally integrated value of 0.2), the polished porous LaB_6_ the intermediate value (0.4) and the rough porous the highest value (0.6). As a term of comparison, it should be noticed that LaB_6_ favourably compares to SiC also if experimental emittances are considered, because the obtained values are significantly lower than those experimentally obtained for SiC pellets (0.9 at 1100 K).

If the spectral shape of emittance curves is concerned, we can observe that for the dense sample, calculated and experimental curves are very similar, while porous samples show some differences. In particular, for porous pellets, we cannot find in experimental emittances the wide bands of local maxima peaked at around 3, 7 and 9 µm wavelengths, that can be seen, on the contrary, in the curves calculated from room temperature spectra. Even if a quantitative evaluation requires additional microstructural measurements after high temperature experiments, which are beyond the scope of this work and will be the subject of a further study, we can explain the obtained results in terms of degassing of impurities still present in porous samples. For instance, according to studies carried out on the boron oxide vapor pressure^45^, 1100 K and ~10^−6^ mbar cause boron oxide species vaporization in boride samples, which could be the case of the LaB6_p sample subjected to high vacuum/high temperature during the emissivity measurement.

In summary, the study we cave carried out both at room and high temperature show that LaB_6_ not only is comparable or better than other boride UHTCs, but in its porous rough form is even better than SiC showing a similar solar absorbance and twice the spectral selectivity, as inferred from room temperature measurements, and a lower measured emittance at 1100 K temperature (0.6 versus 0.9). As already mentioned, SiC is the most advanced solar absorber actually used in plants to date.

## Conclusions

This work is devoted to the study of the spectral reflectance and thermal emittance of pure LaB_6_ and LaB_6_-ZrB_2_-SiC composites in order to evaluate their potential as novel solar absorbers. Pure LaB_6_ materials contained a porosity of about 20%, which resulted in a lower reflectance compared to composite materials. In dense composites containing ZrB_2_ as secondary phase, LaB_6_ has an intrinsic solar radiation selectivity with high reflectance plateau at wavelengths longer than 1 µm and a remarkable α/ε ratio approaching 6.4. Moreover, the porous sample with rough surface favorably compares even to SiC from all points of view, as it shows a similar solar absorbance and a doubled spectral selectivity. Finally, if we consider the thermal emittance at 1000–1100 K temperature, even the most emissive LaB_6_ porous rough sample has a significantly lower emittance than SiC.

However we emphasize that, in our opinion, the innovation potential of LaB_6_ is much higher because, in addition to the promising characteristics listed above, it is also a widely documented thermionic material, and with one of the highest known electron emissivities. Thanks to these properties it could successfully act, at the same time, both as direct high-temperature solar absorber and as electron source, significantly reducing system complexity in future concentrating solar thermionic systems. This could make it a really revolutionary material in solar technology.

## Methods

Commercial powders were used to produce the discs: LaB_6_, (H.C. Starck Grade C), ZrB_2_ (H.C. Starck, grade B), B_4_C (H.C. Starck, grade HS). The monolithic LaB_6_ was produced by hot pressing at 1930 °C, 40 MPa and holding time 15 min. The composite material was obtained by wet ball milling, drying of the starting powders with the following composition:

$$-{{\rm{LaB}}}_{6}+15\,{\rm{vol}} \% \,{{\rm{ZrB}}}_{2}+3\,{\rm{vol}} \% \,{{\rm{B}}}_{4}{\rm{C}}$$

The powder mixture was then densified by hot pressing at 1900 °C, 40 MPa and holding time 15 min. After sintering, the density of the ceramics was experimentally determined by the Archimede method. The sintered pellets were polished using diamond paste up to 1 µm. The samples’ surface were analysed by SEM-EDS (FE-SEM, Carl Zeiss Sigma NTS Gmbh, Oberkochen, DE) and energy dispersive X-ray spectroscopy (EDS, INCA Energy 300, Oxford instruments, UK). The mean surface roughness (*R*_a_) and the distance between the highest asperity, peak or summit, and the lowest valley (*R*_t_) was measured according to the European standard CEN 624-4 using a commercial contact stylus instrument (Taylor Hobson mod. Talysurf Plus) fitted with a 2 µm-radius conical diamond tip over a track length of 8 mm and with a cut-off length of 0.8 mm.

The hemispherical reflectance spectra were acquired using two instruments: a double-beam spectrophotometer (Lambda900 by Perkin Elmer) equipped with a Spectralon®-coated integration sphere for the 0.25–2.5 µm wavelength region and a Fourier Transform spectrophotometer (FT-IR “Excalibur” by Bio-Rad) equipped with a gold-coated integrating sphere and a liquid nitrogen-cooled detector for the range 2.5–16.5 μm.

Thermal emittance has been measured using the setup described in detail in ref. 44 and here briefly recalled for convenience. The apparatus consists of a high-vacuum furnace interfaced either to a Fourier-transform infrared spectrophotometer (Bio-Rad Excalibur) and to a reference blackbody (C.I. Systems SR-2) by means of a splitting optical system. The ultimate pressure limit is few 10^−6^ mbar. The temperature is read on the sample upper surface by means of three thermocouples. The uncertainties are ±20 K on the temperature and ±5% on the spectral emittance. As for the temperature, it should be observed that the furnace heater is able to reach up to 1200 K, but the temperature obtained on the measurement surface of each sample is lower because of losses at thermal contact.
